# Antibodies to myelin oligodendrocyte glycoprotein in optic perineuritis

**DOI:** 10.3389/fimmu.2025.1657600

**Published:** 2025-10-29

**Authors:** Shanshan Cao, Yuan Zhang, Xintong Xu, Mingming Sun, Chunyan Pan, Yuhang Wang, Shihui Wei, Quangang Xu, Huanfen Zhou

**Affiliations:** ^1^ Senior Department of Ophthalmology, Chinese People’s Liberation Army General Hospital & Chinese PLA Medical School, Beijing, China; ^2^ Department of Ophthalmology, Air Force Medical Center of PLA, Beijing, China; ^3^ Department of Ophthalmology, The First Medical Center of Chinese People’s Liberation Army General Hospital & Chinese PLA Medical School, Beijing, China

**Keywords:** optic perineuritis, myelin oligodendrocyte glycoprotein antibody, neuro-ophthalmology, visual prognosis, relapse rate

## Abstract

**Introduction:**

This study aimed to investigate the prevalence of myelin oligodendrocyte glycoprotein antibodies (MOG-IgG) in patients with optic perineuritis (OPN) and to analyze the clinical characteristics and prognosis of MOG-IgG–seropositive cases.

**Methods:**

A retrospective review was conducted of OPN patients diagnosed at the Neuro-ophthalmology Department of the Chinese People’s Liberation Army (PLA) General Hospital between January 2020 and February 2023. Patients were classified into MOG-IgG–positive and seronegative groups based on cell-based assay (CBA) results. Demographic data, clinical manifestations, and visual outcomes were compared between the two groups.

**Results:**

A total of 33 patients (44 eyes) were included, with a mean onset age of 32.7 years (range 6–79) and a male-to-female ratio of 1:2. Bilateral involvement was observed in 33.3% of cases. Eye pain and optic disc swelling were present in 72.7% and 75.8% of patients, respectively. MOG-IgG was detected in 8 patients (24.2%). Compared with seronegative OPN, MOG-IgG-positive patients were significantly younger (mean age 20.8 years vs. 36.6 years, P = 0.034) and had a higher annual relapse rate (median 1.19 vs. 0.31, P = 0.008). All MOG-IgG-positive patients achieved visual acuity (VA) ≥20/40 after the first episode (100.0% vs. 45.5% in seronegative cases, P = 0.005), with this difference persisting at final follow-up (78.6% vs. 47.4%, P = 0.044).

**Discussion:**

These findings highlight distinct clinical characteristics of MOG-IgG-positive OPN, including younger age, higher relapse rates, and superior visual recovery. Detection of MOG-IgG in OPN may facilitate early diagnosis, prognosis prediction, and guide individualized therapeutic strategies.

## Introduction

Optic perineuritis (OPN) represents a rare orbital inflammatory disorder primarily affecting the optic nerve sheath (ONS). The clinical manifestations typically include visual impairment, ocular pain, optic disc swelling, and characteristic visual field abnormalities such as arcuate defects and diffuse peripheral constriction (1). While OPN can emerge as a manifestation of infectious and systemic inflammatory conditions, including syphilis and sarcoidosis (2, 3), the etiology remains idiopathic in the majority of cases. Recent investigations have revealed that myelin oligodendrocyte glycoprotein antibody-associated disease (MOGAD), an immune-mediated demyelinating disorder of the central nervous system (CNS), constitutes the most prevalent underlying etiology of OPN (4).

Myelin oligodendrocyte glycoprotein (MOG) is a component of the myelin sheath in the CNS, mainly expressed on the surface of oligodendrocytes (5, 6). The emergence of MOG-immunoglobulin G (MOG-IgG) has defined a distinct entity, MOG-IgG-associated optic neuritis (MOG-ON), providing new perspectives for the diagnosis and treatment of optic neuritis (ON). In the past few years, MOG-ON has enabled the identification of a subgroup with a clinical course distinct from other optic neuritis phenotypes, highlighting the diagnostic and prognostic value of MOG-IgG testing (7). Recent studies have observed that patients with MOG-IgG-positive ON typically present with visual impairment and have a high propensity for recurrence (8, 9), clinical characteristics that notably overlap with those of OPN. This phenotypic similarity suggests a potentially critical role of MOG-IgG in the pathogenesis of OPN and warrants investigation into the relationship between these conditions.

OPN symptoms are similar to ON but can be differentiated by typical circumferential enhancement of the ONS shown on magnetic resonance imaging (MRI) (10). A retrospective case-control study found that among 48 MOG-ON patients, 31.3% (15/48) exhibited enhancement of ONS on MRI (11). Furthermore, several investigations have suggested a potential association between OPN and MOG-IgG (12–14); however, the literature predominantly consists of isolated case reports lacking systematic analysis of the clinical profiles and prognostic indicators in MOG-IgG–associated OPN (MOG-OPN). This gap in knowledge underscores the importance of the present investigation.

To address this gap, our cohort study aimed to systematically determine the prevalence of MOG-IgG seropositivity in patients diagnosed with OPN and to compare the clinical characteristics and prognostic outcomes between MOG-OPN and MOG-IgG-negative OPN (seronegative OPN) groups. These findings will enhance our understanding of this rare inflammatory condition and potentially guide more targeted therapeutic approaches.

## Materials and methods

### Study design and participants

We conducted a single-center retrospective study at the Department of Neuro-ophthalmology of the Chinese People’s Liberation Army (PLA) General Hospital. All patients diagnosed with OPN between January 2020 and February 2023 were included in the analysis. Serum MOG-IgG testing was performed using a cell-based assay (CBA). Based on MOG-IgG serological status, patients were classified into MOG-OPN and seronegative OPN groups. We collected and compared demographic data, clinical presentations, laboratory findings, treatment protocols, and visual outcomes between the two groups. This study was approved by the Institutional Review Board of the PLA General Hospital (S2021-127-01), and informed consent was obtained from all participants.

### Diagnostic criteria

#### Inclusion criteria

(1) Orbital MRI shows enhancement of the ONS and at least one of the following clinical symptoms: decreased vision, visual field defects, or eye pain; (10, 15)(2)There are follow-up records and the follow-up period is ≥ 24 months.

#### Exclusion criteria

(1) MOG/AQP4 antibody status unknown or AQP4 antibody positive; (2) Orbital MRI shows contrast-enhanced lesions or T2 lesions of the optic nerve; (3) Presence of eye diseases such as glaucoma, anterior segment of the eye, retinal or macular diseases; (4) Any other cause that may lead to systemic inflammation, such as giant cell arteritis, systemic inflammatory diseases, camouflage syndrome and other infectious diseases; (5) Those with incomplete medical history records.

### Study variables and definitions at baseline

In Neuro-ophthalmic examinations, the best corrected visual acuity (BCVA) is the primary outcome, evaluated using a standard visual acuity logarithmic table at a distance of 5 meters. For subjects who cannot read any letters at a distance of 1 meter, further examination will be conducted through finger counting, hand movement, or light perception. The BCVA is evaluated using the Snellen visual acuity chart. Visual acuity measured by BCVA is transformed into the logarithmic minimum angle of resolution (logMAR), calculated as logMAR = -log(BCVA). Specific logMAR values are assigned to qualitative vision assessments: no light perception corresponds to 3.00 logMAR, light perception to 2.70 logMAR, hand motion recognition to 2.00 logMAR, and finger counting to 1.70 logMAR (16). Poor visual recovery is defined as severe visual loss that fails to achieve BCVA ≤ 0.1 (1.0 logMAR) during follow-up. Follow-up visual acuity refers to the BCVA result at least 24 months after the first onset of OPN.

3.0-T orbital MRI with and without gadolinium-based contrast medium was performed on a Discovery 750 scanner (GE Healthcare, Milwaukee, Wisconsin, USA). Each patient underwent the same imaging protocol, which consisted of pre-contrast axial T1-weighted scans (TR/TE: 1750/24 ms) alongside axial and coronal T2-weighted sequences (TR/TE: 4248/100 ms and 6802/100 ms, respectively). Following this, post-contrast T1-weighted images were acquired in both axial and coronal orientations, with a slice thickness of 3 mm and a 0.5 mm gap between slices. An intravenous injection of gadolinium-diethylenetriamine pentaacetic acid was administered at a standard dosage of 0.1 mmol per kilogram of body weight. After contrast administration, enhanced T1-weighted images in the axial, sagittal, and coronal planes were obtained. Two experienced neuro-ophthalmologists and one neuroradiologist conducted a blind and independent evaluation of the orbital MRI.

The peripapillary retinal nerve fiber layer (pRNFL) was assessed using spectral-domain optical coherence tomography (SD-OCT; Carl Zeiss Meditec, Dublin, CA, USA). All scans were performed by a trained technician according to a standardized protocol. pRNFL thickness was measured in optic disc mode, employing a 6 × 6 mm scan area with a circular scan (diameter: 3.4 mm) centered on the optic disc. The device**’**s built-in software automatically segmented the pRNFL boundaries, and both the average pRNFL thickness and quadrant-specific values (superior, inferior, nasal, and temporal) were recorded.

The definition of clinical recurrence is the recurrence of clinical attacks at least 30 days after the last onset (17). The types of onset include unilateral and bilateral OPN.

### Laboratory examinations

CBA using indirect immunofluorescence was employed to detect serum MOG and aquaporin 4 (AQP4) IgG. Serum samples were deemed seropositive if the MOG-IgG titer was 1:100 or higher.

Serum samples from all patients were tested for autoantibodies, including antinuclear antibodies (ANA), anti-Sjogren’s syndrome associated antigen A (SSA), anti-Sjogren’s syndrome associated antigen B (SSB), human leukocyte antigen B27 (HLA-B27), anti-thyroglobulin antibodies (ATAs), anti-thyroid peroxidase autoantibodies (anti-TPOAb), anticardiolipin antibodies (ACLs and β2-GPI), anti-centromeric antibodies (ACA), anti-neutrophil cytoplasmic antibodies (ANCA), rheumatoid factor (RF), and anti-perinuclear factor (APF).

All patients underwent screening for infectious etiologies, including tuberculosis (TB), syphilis, human immunodeficiency virus (HIV), and other common pathogens, prior to enrollment. TB screening consisted of chest CT, interferon-gamma release assay (IGRA), and review of clinical history. None of the enrolled patients showed evidence of active or latent TB, nor of infection with other viral antigens.

Cerebrospinal Fluid (CSF) variables were evaluated using data from the results of the lumbar puncture in the acute onset of optic perineuritis. The CSF was tested for white blood cell count, protein, and IgG levels. If the white blood cell count is greater than 10/μL, the protein level is greater than 400mg/L, and the IgG level is greater than 3.4 pg/mL, it is considered that the measurement value has increased.

### Statistical analysis

Statistical analysis and data processing were performed using SPSS 27.0 and R (version 4.2.1). Normally distributed continuous data are expressed as Mean ± SD, while two-group comparisons use independent samples t-tests, and Skewed continuous data are expressed as median (interquartile range [IQR]), analyzed using the Mann-Whitney test for independent samples. For OCT pRNFL data, a Generalized Estimating Equation (GEE) model was used to compare differences between groups. For categorical data expressed as n (%), the chi-square test or Fisher’s exact test is used for inter-group comparison; All tests are two-sided tests, with a test level of α=0.05 and a P value less than 0.05 considered statistically significant.

## Results

### Demographics and clinical characteristics

**Figure 1:**
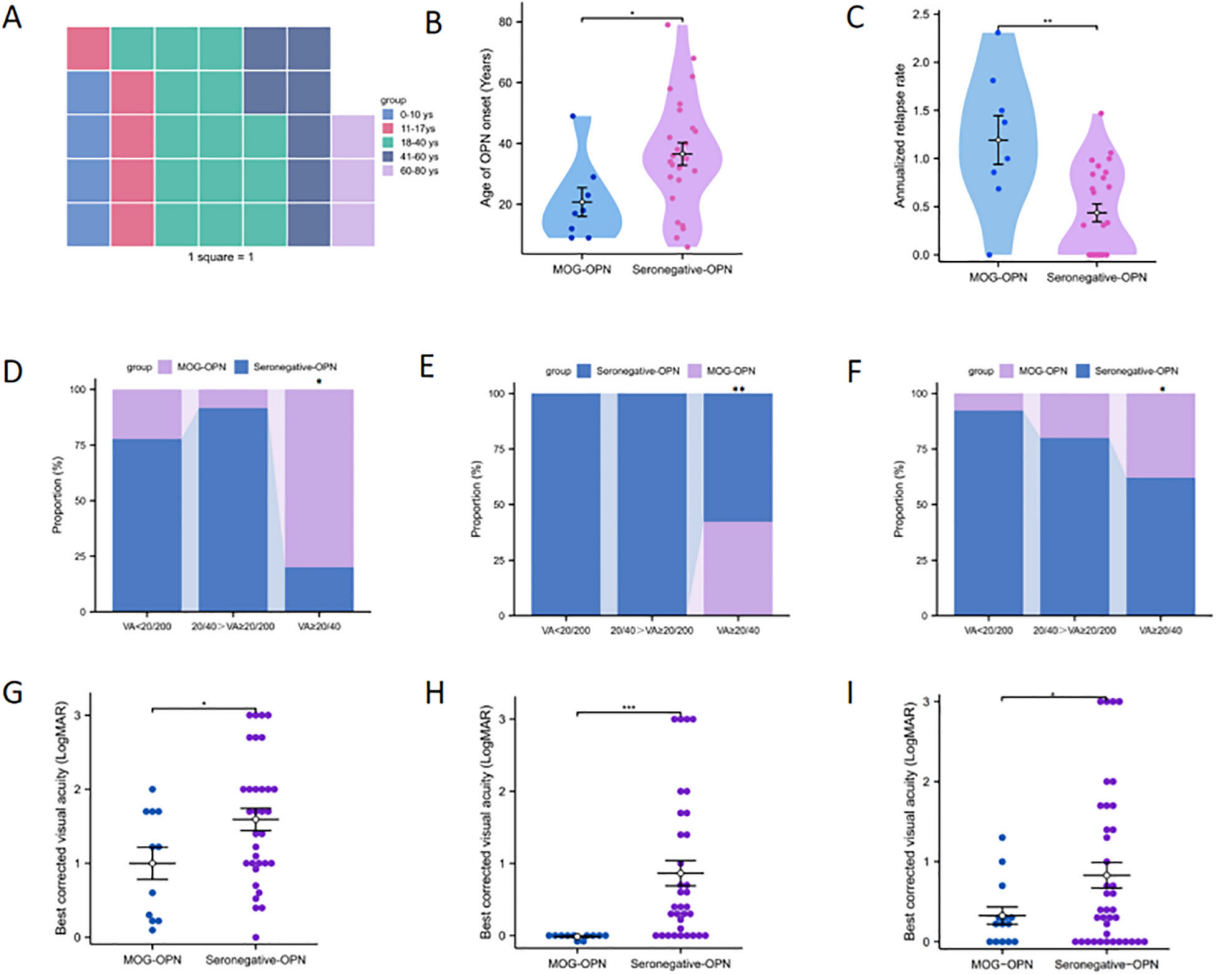
Demographic and clinical characteristics of patients with optic perineuritis (OPN). **(A)** The waffle plot displays the age distribution of OPN patients. **(B)** Age distribution of OPN subtypes based on myelin oligodendrocyte glycoprotein antibody (MOG Abs) status. **(C)** Annual recurrence rate (ARR) among OPN subtypes. **(D-F)** respectively show the proportion of patients with varying degrees of visual impairment at disease onset, after first-episode recovery, and at last follow-up, in both groups. **(G-I)** respectively show the LogMAR in the two groups at disease onset, after first-episode recovery, and at the last follow-up. *P < 0.05; **P < 0.01; ***P < 0.001.

**Figure 2:**
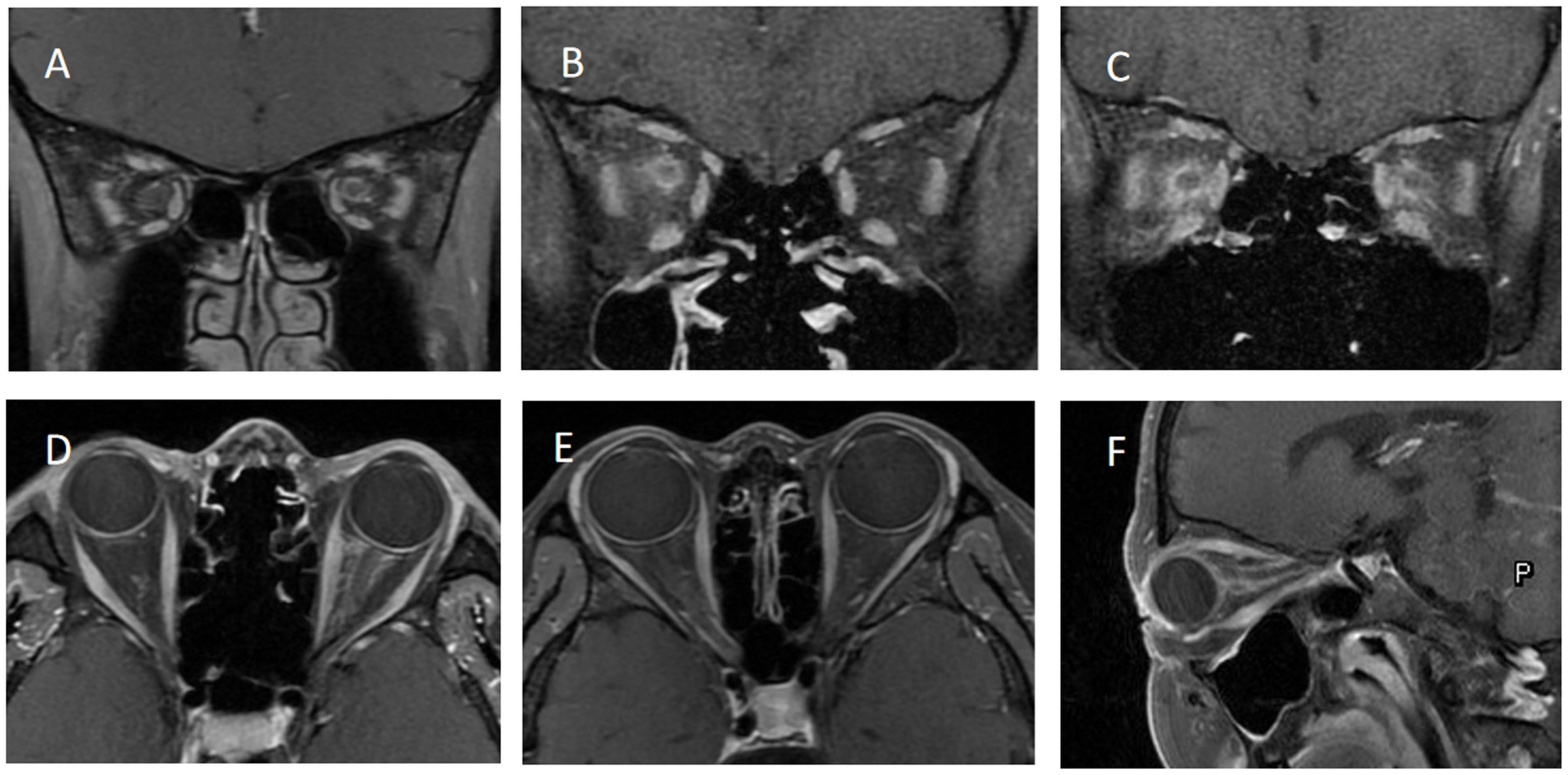
shows the orbital MRI of different optic perineuritis (OPN) patients. **(A)** The coronal T1 contrast image showed enhancement of the left optic nerve sheath (ONS). **(B)** Coronal T1 contrast image showed enhancement of the right ONS **(C)** Coronal T1 contrast image showed enhancement of both ONS. **(D)** Axial T1 contrast image showed enhancement of left ONS. **(E)** Axial T1 contrast image showed enhancement of right ONS. **(F)** The sagittal T1 contrast image showed left ONS.

**Table 1** provides a comprehensive overview of the demographic and clinical characteristics of MOG-ON cases at the time of onset. This study evaluated a total of 33 patients (44 eyes), with 24.2% testing positive for MOG-IgG in their serum, including 8 cases (24.2%) of MOG-OPN and 25 cases (33.3%) of Seronegative OPN; The average age of onset of OPN in this study cohort was 32.73 ± 18.61 years (age range 6–79 years), of which 24 cases (72.7%) were adults (≥ 18 years) (**Figure 1A**), 22 cases (66.7%) were females, 11 cases (33.3%) showed bilateral involvement, 24 cases (72.7%) had eye pain and 25 cases (75.8%) had optic disc swelling at the first onset, 7 cases (21.2%) had a fever or respiratory infections before the onset of OPN. The average time from onset to the worst vision was 8.33 ± 4.29 days. 32 patients (97.0%) had different visual field abnormalities. All patients showed ONS enhancement in orbital MRI, and **Figure 2** showed the classic ONS enhancement in different patients.

**Table 1:** The demographic and clinical characteristics in this cohort of OPN patients.

Characteristics	OPN	Percentage
No. of patients (eyes)	33 (44)	
Age at onset, mean ± SD, y	32.73 ± 18.61	
Adults (≥18 years)	24	72.7
Children (<18 years)	9	27.3
Sex		
Male	11	33.3
Female	22	66.7
Phenotype at onset		
Unilateral OPN	22	66.7
Bilateral OPN	11	33.3
Antibodies serostatus		
MOG-Ab	8	24.2
Double antibodies negative	25	75.8
Ocular pain	24	72.7
Optic disc swelling	25	75.8
Visual field defect, eyes, n	32	
Arcuate scotoma	13	40.6
Diffuse visual field defect	8	25.0
Irregular defect	7	21.9
Central scotoma	2	6.3
Blind spot enlargement	2	6.3
Duration from attack to nadir, mean mean ± SD, days	8.33 ± 4.29	
Previous infection, n (%)	7	21.2
BCVA at first onset, eyes, n	44	
VA<20/200	27	61.4
20/40>VA≥20/200	12	27.3
VA≥20/40	5	11.4
Mean ± SD (logMAR)	1.44 ± 0.86	
BCVA recovery at the first onset, eyes, n	44	
VA<20/200	10	22.7
20/40>VA≥20/200	8	18.2
VA≥20/40	26	59.1
Mean ± SD (logMAR)	0.64 ± 0.95	
BCVA at last follow-up, eyes, n	52	
VA<20/200	13	25.0
20/40>VA≥20/200	10	19.2
VA≥20/40	29	55.8
Mean ± SD (LogMAR)	0.69 ± 0.89	
Bilateral OPN at last follow-up, n	19	57.6
OPN recurrence within one year, n	9	27.3
OPN recurrence within two years, n	17	51.5
ARR, median, (IQR)	0.69 (0.00, 0.98)	
Treatment		
IVMP	28	84.8
Oral prednisolone	5	15.2
Mycophenolate	4	12.1
Rituximab	3	9.1
Azathioprine	2	6.1
Follow-up, mean ± SD	39.91 ± 10.61	

BCVA, best corrected visual acuity; ARR, annualized relapse rate; IV, intravenous;IVMP, intravenous methylprednisolone.

In this study, 27 eyes (61.4%) experienced severe vision loss (visual acuity <20/200) during the first episode, and 29 eyes (55.8%) had good vision recovery (≥20/40) at the last visit. The mean follow-up was 39.91 ± 10.61 months. By the last follow-up, 19 patients (57.6%) had bilateral involvement. Within the first year after OPN onset, 9 patients (27.3%) relapsed, and by the second year, 17 patients (51.5%) had experienced a relapse. The treatment of these patients is listed in **Table 1**.

Patients were divided into two groups based on the results of serological MOG-antibody testing, 8 (24.2%) were MOG-IgG seropositive. The clinical characteristics of the two groups of patients were compared (**Table 2**). The mean age was lower in the MOG-OPN group (20.75 ± 13.34 years) than in the seronegative OPN group (36.56 ± 18.62 years; P = 0.034) (**Figure 1B**). Time to nadir was shorter in the MOG-OPN group (5.88 ± 4.26 days vs. 9.12 ± 4.08days; P = 0.061), and the median ARR was higher in the MOG-OPN group(1.19 [IQR, 0.81-1.58] vs. 0.31 [0.00-0.84]; P = 0.008) (**Figure 1C**). Follow-up duration did not differ between groups (42.38 ± 14.39 vs. 39.12 ± 9.33 months; P = 0.459).

**Table 2:** The clinical characteristics and visual recovery in different sub-groups of OPN patients.

Characteristics	MOG-OPN	Seronegative OPN	P value
No. patients	8	25	
Mean age at onset, years (mean ± SD)	20.75 ± 13.34	36.56 ± 18.62	0.034*
Female, n (%)	7 (87.5)	15 (60.0)	0.218
Bilateral ON at onset, n (%)	3 (37.5)	8 (32.0)	1.000
Ocular pain, n (%)	7 (87.5)	17 (68.0)	0.394
Optic disc swelling, n (%)	6 (75.0)	19 (76.0)	1.000
Previous infection, n (%)	5 (62.5)	2 (8.0)	0.004**
Abnormal autoimmune antibodies, n (%)	1 (12.5)	1 (4.0)	0.432
Duration from attack to nadir, mean mean ± SD, days	5.88 ± 4.26	9.12 ± 4.08	0.061
BCVA at first onset, eyes, n	11	33	
VA<20/200	6 (54.5)	21 (63.6)	0.858
20/40>VA≥20/200	1 (9.1)	11 (33.3)	0.241
VA≥20/40	4 (36.4)	1 (3.0)	0.014*
Mean ± SD (logMAR)	1.00 ± 0.72	1.59 ± 0.86	0.046*
BCVA recovery at first onset, eyes, n	11	33	
VA<20/200	0 (0.0)	10 (30.3)	0.097
20/40>VA≥20/200	0 (0.0)	8 (24.2)	0.176
VA≥20/40	11 (100.0)	15 (45.5)	0.005**
Mean ± SD (logMAR)	-0.01 ± 0.03	0.86 ± 1.01	<.001***
BCVA at last follow-up, eyes, n	14	38	
VA<20/200	1 (7.1)	12 (31.6)	0.149
20/40>VA≥20/200	2 (14.3)	8 (21.1)	0.879
VA≥20/40	11 (78.6)	18(47.4)	0.044*
Mean ± SD (logMAR)	0.33 ± 0.40	0.83 ± 0.98	0.012*
Bilateral ON at last follow-up, n (%)	6 (75.0)	13 (52.0)	0.416
ON recurrence within one year, n (%)	2 (25.0)	7 (28.0)	1.000
ON recurrence within two years, n (%)	6 (75.0)	11 (44.0)	0.432
ARR, median, (IQR)	1.19 (0.81, 1.58)	0.31 (0.00, 0.84)	0.008**
Follow-up, mean ± SD	42.38 ± 14.39	39.12 ± 9.33	0.459

BCVA, best corrected visual acuity;IQR, interquartile range; *P<0.05, **P<0.01, ***P<0.001.

The MOG-OPN group had a higher proportion of participants with previous infection (62.5% vs. 8.0%; P = 0.004), and mycophenolate mofetil use (37.5% vs. 4.0%; P = 0.036). No significant differences were observed in sex distribution, bilateral involvement, ocular pain, optic disc swelling, or recurrence rates within one or two years (P > 0.05 for all).

### Visual outcome, clinical prognosis, and relapsing characteristics

We compared the visual outcomes, clinical prognosis, and annual relapse rate between the MOG-OPN group and the seronegative OPN group (**Table 2**). Among 44 eyes, 11 (25.0%) were classified as MOG-OPN group and 33 (75.0%) as seronegative OPN group. For BCVA at first onset, no significant between-group differences were observed in the proportion of eyes with visual acuity (VA) <20/200 (54.5% in MOG-OPN group vs. 63.6% in seronegative OPN group; P = 0.858) or VA between 20/40 and 20/200 (9.1% vs. 33.3%; P = 0.241). However, the MOG-OPN group had a higher proportion of eyes with VA≥20/40 (36.4% vs. 3.0%; P = 0.014) (**Figure 1D**). Mean LogMAR scores were lower in the MOG-OPN group (1.00 ± 0.72) than in the seronegative OPN group (1.59 ± 0.86; P = 0.046) (**Figure 1G**). For BCVA recovery at first onset, no significant differences were observed in the proportion with VA <20/200 (0% in MOG-OPN group vs. 30.3% in seronegative OPN group; P = 0.10) or VA between 20/40 and 20/200 (0% vs. 24.2%; P = 0.18). However, all participants in the MOG-OPN group achieved VA≥20/40 (100.0%), compared with 45.5% in the seronegative OPN group (P = 0.005) (**Figure 1E**). Similarly, mean LogMAR scores were lower in the MOG-OPN group (−0.01 ± 0.03) compared with the seronegative OPN group (0.86 ± 1.01; P < 0.001) (**Figure 1H**).

At the last follow-up, 19 patients had bilateral involvement, totaling 52 eyes. Among 52 eyes, 14 (26.9%) were classified as MOG-OPN and 38 (73.1%) as seronegative OPN group. The mean LogMAR was higher in the seronegative OPN group (0.83 ± 0.98) than in MOG-OPN (0.33 ± 0.40; P = 0.012) (**Figure 1I**). Severe visual impairment (VA <20/200) was present in 13 participants (25.0%), with no significant between-group difference (7.1% in MOG-OPN vs. 31.6% in seronegative OPN group; P = 0.149). Moderate-to-severe visual impairment (20/40>VA≥20/200) was observed in 10 participants (19.2%), with no significant difference between groups (P = 0.879). A higher proportion of participants in MOG-OPN (78.6%) had VA ≥20/40 compared with the seronegative OPN group (47.4%; P = 0.044) (**Figure 1F**).

### CSF analysis and OCT measurements

**Table 3** shows the comparison of CSF analysis components and pRNFL thickness between the two groups. 8 (26.7%) MOG-OPN patients and 22 (73.3%) seronegative OPN patients underwent lumbar puncture. A higher proportion of participants in the MOG-OPN group had abnormal CSF WBC counts (37.5% vs. 4.5%; P = 0.048). No statistically significant differences were observed in CSF protein abnormality (25.0% vs. 36.4%; P = 0.682) or CSF IgG abnormality (37.5% vs. 36.4%; P = 1.000).

**Table 3:** Comparison of CSF and pRNFL thickness between MOG-OPN and seronegative OPN groups.

Characteristics	MOG-OPN	Seronegative OPN	P value
No.of patients	8	25	
No. of eyes	11	33	
No. of lumbar punctures	8	22	
CSF white cell count, mean ± SD (n/mm3)	6.00 ± 6.61	1.95 ± 3.36	0.135
White cell counts elevated, n(%)	3 (37.5)	1 (4.5)	0.048*^†^
CSF protein, mean ± SD (g/L)	270.86 ± 99.96	366.89 ± 132.27	0.073
Protein elevated, n(%)	2 (25.0)	8 (36.4)	0.682^†^
CSF IgG level, mean ± SD (mg/dL)	2.16 ± 1.20	2.81 ± 1.34	0.236
IgG level elevated, n(%)	3 (37.5)	8 (36.4)	1.000^†^
pRNFL (μm), eyes, n	10	26	
Average thickness (mean ± SD)	77.60 ± 3.57	76.46 ± 2.92	0.757‡
Superior quadrant (mean ± SD)	100.00 ± 6.79	92.85 ± 5.26	0.403‡
Inferior quadrant (mean ± SD)	95.60 ± 5.05	93.31 ± 4.73	0.732‡
Nasal quadrant (mean ± SD)	63.90 ± 2.96	63.38 ± 2.19	0.768‡
Temporal quadrant (mean ± SD)	49.90 ± 4.05	55.50 ± 1.67	0.187‡

^†^Fisher exact; ‡ Generalized Estimating Equation (GEE) Analysis; CSF, cerebrospinal fluid; pRNFL, peripapillary retinal nerve fiber layer; *P<0.05.

The mean (± SD) average thickness with no significant difference between MOG-OPN group (77.60 ± 3.57) and Seronegative OPN group (76.46 ± 2.92; P = 0.757). Similarly, no statistically significant differences were observed in the superior quadrant (100.00 ± 6.79 vs. 92.85 ± 5.26; P = 0.403), inferior quadrant (95.60 ± 5.05 vs. 93.31 ± 4.73; P = 0.732), nasal quadrant (63.90 ± 2.96 vs. 63.38 ± 2.19; P = 0.768), or temporal quadrant (49.90 ± 4.05 vs. 55.50 ± 1.67; P = 0.187).

## Discussion

OPN is a heterogeneous condition primarily characterized by inflammation of the ONS (18). ONS enhancement represents a common radiological finding in patients with MOG-ON, occurring in approximately 30%-52% of cases (19, 20). However, the prevalence of MOG-IgG specifically among OPN patients has not been systematically investigated. In our single-center retrospective study of 33 confirmed OPN cases, we found that 24.2% (8/33) exhibited MOG-IgG seropositivity. To our knowledge, this represents the first systematic analysis of MOG-IgG seroprevalence in OPN patients, and further characterizes the distinct clinical features and prognostic indicators of MOG-OPN in a Chinese cohort.

A retrospective case-control study found that among 48 MOGAD patients, 31.3% (15/48) showed ONS enhancement on MRI (11), suggesting a radiological overlap between MOGAD and OPN. Furthermore, in a Canadian case series examining the etiology of secondary OPN, MOGAD was identified as the most common underlying cause, with 26.7% (4/15) of patients testing positive for MOG-IgG. This study also revealed that patients with ONS enhancement and those with idiopathic OPN presented with comparable clinical manifestations (4).It is plausible that MOGAD may account for a substantial proportion of cases previously classified as idiopathic OPN in earlier literature, considering that MOGAD as a distinct clinical entity has only been fully characterized in recent years. Given these findings, MOG-IgG testing using CBA, which has been widely validated and implemented, should be incorporated into the standard diagnostic algorithm for OPN of undetermined etiology (21–23).

Our analysis identified distinct clinical characteristics of OPN in a Chinese patient cohort. Demographically, the cohort demonstrated a female predominance with a male-to-female ratio of 1:2 and a mean age at onset of 32.7 years. Clinically, the predominant presenting symptoms included ocular pain and optic disc swelling, while arcuate scotoma represented the most common visual field defect—findings consistent with the 2001 study by Purvin et al. (10) Notably, 57.6% of patients developed bilateral involvement during the disease course, and 51.5% experienced at least one relapse during the 2-year follow-up period. These outcomes differ from previous reports (24), possibly because our study specifically evaluated both idiopathic OPN and MOG-IgG-seropositive OPN patients, whereas earlier investigations predominantly included secondary OPN cases without MOG-IgG testing.

In this study, we observed differences between the MOG-OPN group and the seronegative OPN group. The mean age at onset was significantly lower in the MOG-OPN group (20.75 ± 13.34 years) than in the seronegative OPN group (36.56 ± 18.62 years; P = 0.034). The median annualized relapse rate (ARR) was higher in the MOG-OPN group (1.19 [IQR, 0.81-1.58] vs. 0.31 [0.00-0.84]; P = 0.008). These findings suggest that MOG-OPN patients are predominantly young and often present with recurrent courses, which may assist in early diagnosis and the implementation of timely therapeutic interventions.

MOG-IgG can be detected in AQP4-IgG negative neuromyelitis optica spectrum disorder and ON, and some of these patients will develop a recurrent form of the disease (25, 26). In our cohort, 75.0% of patients in the MOG-OPN group relapsed within two years. Studies indicate that persistent MOG-IgG titers are strongly associated with disease relapse (27, 28), necessitating long-term close monitoring and personalized treatment strategies for these patients. However, due to limited repeated MOG-OPN testing, we couldn’t establish the relationship between MOG-IgG titers and disease recurrence. Overall, MOG antibodies, as emerging biomarkers, open new avenues for the clinical management of OPN. However, additional studies are required to clarify the core processes and refine therapeutic methods.

Our research indicates that after the first attack, all 11 eyes (100.0%) in the MOG-OPN group achieved good visual recovery (VA ≥ 20/40). The LogMAR was superior to the seronegative OPN group (-0.01 ± 0.03 vs 0.86 ± 1.01; P < 0.001). At the last follow-up, 11 eyes (78.6%) in the MOG-OPN group retained good vision, with LogMAR still better than the seronegative OPN group (0.33 ± 0.40 vs 0.83 ± 0.98; P = 0.012). This suggests that MOG-OPN patients are more likely to have better visual outcomes. This aligns with prior MOG-ON findings, where 98% of MOG-ON patients achieved functional vision of ≥ 20/40 after acute treatment (29), implying MOG antibodies may influence disease progression and prognosis.

The underlying biological mechanisms may help explain these findings. MOG, located on the outermost surface of the myelin sheath, is readily accessible to circulating autoantibodies. Binding of MOG-IgG can activate the complement cascade and trigger antibody-dependent cellular cytotoxicity, leading to demyelination and inflammatory infiltration (30). In MOG-OPN, such immune-mediated injury may extend to the perineural sheath via contiguous inflammation and disruption of the blood-nerve barrier, resulting in the characteristic perineural enhancement observed, which may account for the favorable visual prognosis.

Our study showed that a higher proportion of the MOG-OPN group had abnormal CSF WBC counts (37.5% vs. 4.5%; P = 0.048) and the MOG-OPN group had a higher proportion of participants with previous infections (62.5% vs. 8.0%; P = 0.004), which suggests a more pronounced inflammatory response *in vivo*. The association between MOG-IgG and prior infections may indicate that previous infections could be a potential trigger for MOG-OPN. This aligns with previous studies on MOG-ON, where approximately 37.5% to 67% of MOG antibody-positive patients had a history of infection before onset (31, 32). These infections may trigger immune responses against MOG antigens through various mechanisms, including molecular mimicry, bystander activation, or epitope spreading. For example, direct CNS infections may lead to CNS antigens entering the peripheral circulation, activating brain-derived T cells, and subsequently inducing autoimmune responses against MOG. Additionally, infections may disrupt the blood-brain barrier, exposing MOG antigens to the peripheral immune system and activating B cells to produce MOG antibodies. These findings provide valuable insights into the clinical and inflammatory characteristics of MOG-OPN, highlighting its distinct features compared to seronegative OPN.

Our study revealed severe pRNFL atrophy in OPN patients, a finding consistent with a previous study that also demonstrated significant RNFL atrophy in OPN patients (4). However, we found no difference in pRNFL atrophy between the MOG-OPN group and the seronegative OPN group.

This study has several limitations. First, the single-center design and relatively small sample size may have introduced selection bias, reduced statistical power, and limited the generalizability of our findings to broader populations. Second, the restricted demographic diversity further constrains extrapolation to other ethnic or geographic groups. Third, the retrospective nature of this investigation precludes standardized follow-up protocols and comprehensive longitudinal data collection. Finally, the absence of serial MOG-IgG measurements in all patients prevented assessment of potential correlations between antibody titer dynamics and clinical outcomes or treatment responses. Future studies should address these limitations by adopting multicenter, prospective designs with larger and more demographically diverse cohorts. Extended follow-up is warranted to allow a more accurate characterization of recurrence patterns, long-term visual outcomes, and the prognostic implications of MOG-IgG seroprevalence in OPN.

In this first systematic investigation of MOG-IgG seropositivity among patients with OPN in China, we found a seroprevalence of 24.2%. Our comparative analysis revealed that MOG-IgG-positive OPN patients display distinct clinical characteristics, including a significantly younger age at onset, predominantly bilateral involvement, better visual recovery following treatment, and a notably higher relapse rate compared to seronegative OPN patients. These distinctive features suggest that MOG-OPN represents a clinically relevant immunopathogenic subtype of OPN rather than merely a coincidental association.

## Data Availability

The raw data supporting the conclusions of this article will be made available by the authors, without undue reservation.
